# Challenges and strategies in patients’ health priorities-aligned decision-making for older adults with multiple chronic conditions

**DOI:** 10.1371/journal.pone.0218249

**Published:** 2019-06-10

**Authors:** Mary Tinetti, Lilian Dindo, Cynthia Daisy Smith, Caroline Blaum, Darce Costello, Gregory Ouellet, Jonathan Rosen, Kizzy Hernandez-Bigos, Mary Geda, Aanand Naik

**Affiliations:** 1 Department of Medicine, Yale School of Medicine, New Haven, Connecticut, United States of America; 2 Department of Chronic Disease Epidemiology, Yale School of Public Health, New Haven, Connecticut, United States of America; 3 Houston Center for Innovations in Quality, Effectiveness and Safety, Michael E. DeBakey VA Medical Center, Texas, United States of America; 4 Menninger Department of Psychiatry and Behavioral Sciences, Baylor College of Medicine, Houston, Texas, United States of America; 5 American College of Physicians, Philadelphia, Pennsylvania, United States of America; 6 Perelman School of Medicine at the University of Pennsylvania, Philadelphia, Pennsylvania, United States of America; 7 Department of Internal Medicine, New York University School of Medicine, New York City, New York, United States of America; 8 Connecticut Center for Primary Care, Hartford, Connecticut, United States of America; 9 Alkek Department of Medicine, Baylor College of Medicine, Houston, Texas, United States of America; University of Malaya, MALAYSIA

## Abstract

**Objectives:**

While patients’ health priorities should inform healthcare, strategies for doing so are lacking for patients with multiple conditions. We describe challenges to, and strategies that support, patients’ priorities-aligned decision-making.

**Design:**

Participant observation qualitative study.

**Setting:**

Primary care and cardiology practices in Connecticut.

**Participants:**

Ten primary care clinicians, five cardiologists, and the Patient Priorities implementation team (four geriatricians, physician expert in clinician training, behavioral medicine expert). The patients discussed were ≥ 66 years with >3 chronic conditions and ≥10 medications or saw ≥ two specialists.

**Exposure:**

Following initial training and experience in providing Patient Priorities Care, the clinicians and Patient Priorities implementation team participated in 21 case-based, group discussions (10 face-to-face;11 telephonic). Using emergent learning (i.e. learning which arises from interactions among the participants), participants discussed challenges, posed solutions, and worked together to determine how to align care options with the health priorities of 35 patients participating in the Patient Priorities Care pilot.

**Main outcomes:**

Challenges to, and strategies for, aligning decision-making with patient’s health priorities.

**Results:**

Categories of challenges discussed among participants included uncertainty, complexity, and multiplicity of problems and treatments; difficulty switching to patients’ priorities as the focus of decision-making; and differing perspectives between patients and clinicians, and among clinicians. Strategies identified to support patient priorities-aligned decision-making included starting with one thing that matters most to each patient; conducting serial trials of starting, stopping, or continuing interventions; focusing on function (i.e. achieving patient’s desired activities) rather than eliminating symptoms; basing communications, decision-making, and effectiveness on patients’ priorities not solely on diseases; and negotiating shared decisions when there are differences in perspectives.

**Conclusions:**

The discrete set of challenges encountered and the implementable strategies identified suggest that patient priorities-aligned decision-making in the care of patients with multiple chronic conditions is feasible, albeit complicated. Findings require replication in additional settings and determination of their effect on patient outcomes.

## Introduction

Healthcare decision-making for persons with multiple chronic conditions (MCCs) is difficult [1–5]. The focus on managing individual conditions fails to account for interactions among multiple conditions and their treatments, leading to uncertain benefit and potential harm [3–6]. Evidence to guide care is often lacking because individuals with MCCs are excluded from most clinical trials [7–8]. Even trials that include these individuals address disease-specific outcomes or survival, not always the outcomes most valued by older adults with MCCs [2]. The number and complexity of patient-related activities such as medications, testing, health visits, and self-monitoring tasks, are increasingly burdensome [2,9–12]. Older adults with multiple chronic conditions, when faced with tradeoffs that require difficult choices, vary in their health outcome goals and in their preferences for the healthcare they are willing and able to receive [12–15].

There is consensus that healthcare should be, “*respectful of and responsive to individual patient preferences*, *needs*, *and values and ensuring that patient values guide all clinical decisions*” [16]. Communication strategies facilitate patient preferences—and priorities—based decision-making for persons with serious illness or near the end-of-life [17–20]. However, methods for ascertaining the health priorities of older adults with multiple conditions who are not near the end-of life remain lacking, as do reliable approaches for clinicians wishing to align decision-making and care with these priorities [1,2,12,13].

**Fig 1:**
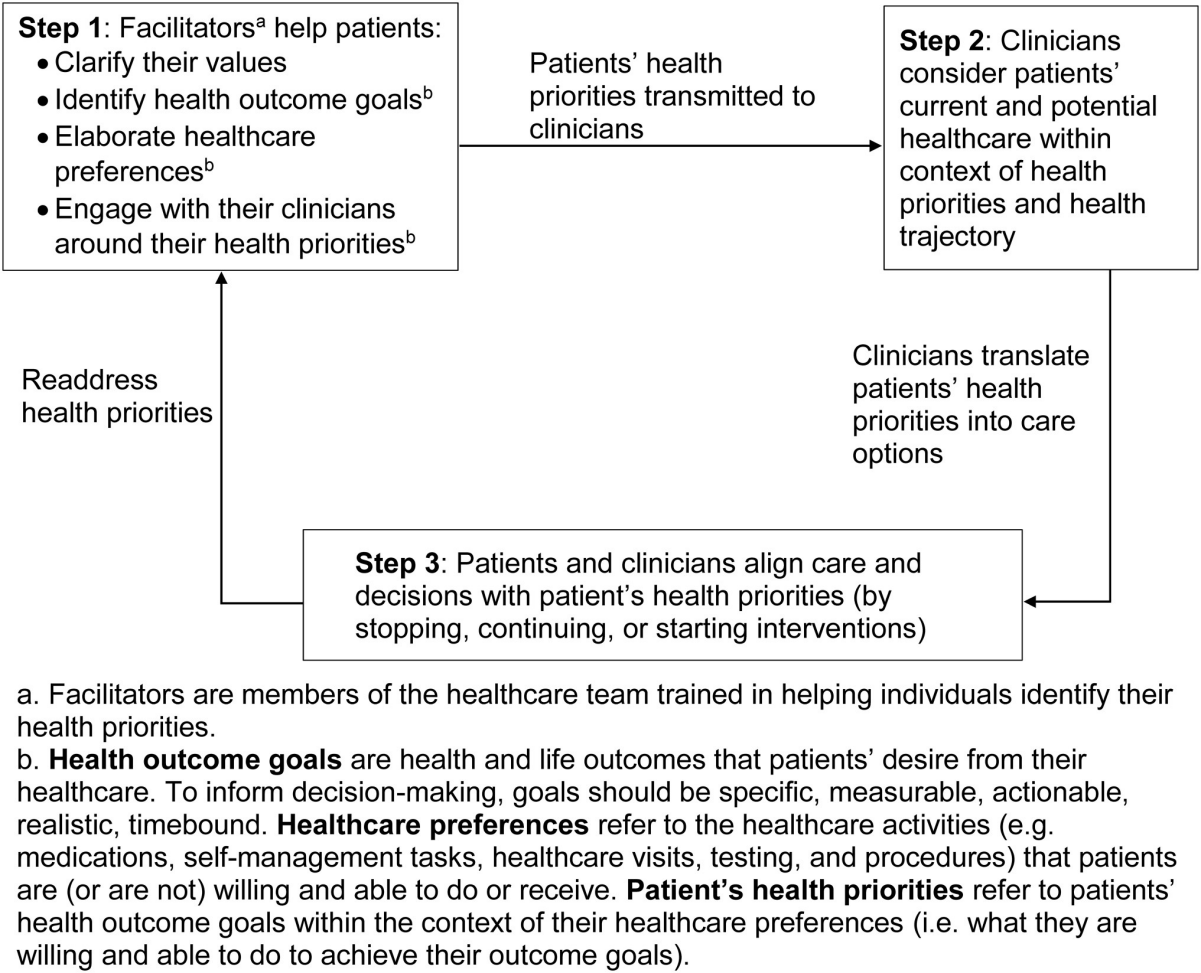
Steps in Patient Priorities Care.

To fill these gaps, we launched the Patient Priorities Care initiative with input from clinicians, patients, caregivers, healthcare system representatives, health information technology and redesign experts, and payers [21]. This diverse group of stakeholders agreed that identifying and aligning decision-making with each patient’s health priorities was the approach that best addressed the uncertainty and treatment burden inherent in the care of multiple conditions while honoring the directive to ensure, “…*that patient values guide all clinical decisions*.” [15,21]. Based on this input, we designed a prototype, outlined in Fig 1, that aligns decision-making and care with each patient’s health priorities, namely their health outcome goals and healthcare preferences (see Fig 1 for definitions) [21,22]. As described previously, Patient Priorities Care is a continuous process that begins when patients—and family members or friends when desired by patients—identify specific, actionable, realistic, and reliable health priorities facilitated by a member (e.g. nurse, advanced practice nurse, social worker) of the healthcare team (**Step 1** in Fig 1) [23]. These health priorities are transmitted to clinicians who use them in their communication and decision-making with patients and other clinicians (**Steps 2** and **3**). We recently described the feasibility of implementing Patient Priorities Care in practice [24].

Effective and feasible strategies for translating disease-specific into priorities-aligned care options and addressing the challenges inherent in patient health priorities aligned decision-making are essential to clinician participation in this approach. To help identify these strategies, we sought insights into how clinicians might link patients’ priorities to decision-making (**Steps 2** and **3**). The first aim was to describe challenges the clinicians faced in trying to align clinical decisions with patients’ health priorities. The second aim was to identify strategies that emerged to help clinicians overcome challenges to providing patient priorities-aligned care.

## Methods

### Design

We used a qualitative participant observation design in which investigators observed, participated in, and interpreted discussions with clinicians [25].

### Setting

This work involved the primary care and cardiology practices in Connecticut participating in development and testing of Patient Priorities Care [23,24].

### Participants

The Patient Priorities implementation team included four geriatricians, a general internist expert in clinician training, an expert in behavioral medicine, the clinical champion from the primary care practice, two priorities facilitators (an Advanced Practice Nurse (APN) and a care manager employed by the primary care practice who helped patients identify their health priorities), and two project managers. The practicing clinicians who participated in the pilot included the ten primary care providers (three APNs, one physician assistant, and five physicians) who worked at the pilot primary care practice and the five cardiologists who provided most of the cardiology care for patients from the pilot primary care practice. All primary care clinicians and cardiologists providing care at these practices participated in the pilot. The primary care practice is the largest clinical site of a multi-site primary care group practice providing care to nearly 15% of people in Connecticut. We included cardiologists because they are responsible for a large amount of the specialty care received by older adults with MCCs. The selection and recruitment of the pilot practice and the participating clinicians were described previously [24]. The clinicians received modest stipends for participating in Patient Priorities Care.

The primary care clinicians and cardiologists were introduced to the concepts of Patient Priorities Care through an introductory webinar; they then participated in two case-based face-to-face training sessions in September and October 2016 [24]. During these training sessions they role played patient-clinician and clinician-clinician scenarios involving commonly encountered decisional issues for older adults with multiple chronic conditions. From November 2016-February 2017, geriatrician members of the Patient Priorities team (MT, CB, GO) met monthly with the PCPs and every other month with the cardiologists to discuss the workflow involved in Patient Priorities Care, challenges encountered, and decisions or changes in care that occurred from knowing patients’ health priorities.

### Exposure

Following the initial training and experience in providing Patient Priorities Care, the participating clinicians and the Patient Priorities implementation team participated in 21 group discussions between March 2017- March 2018. Participants received a summary of the health conditions, medications, recent procedures, functional status, and health priorities template of 1–2 patients participating in the pilot selected by the priorities facilitators (example of a template in S1 File). The template included the patient’s health values, health outcome goals, healthcare preferences, self-perception of their health trajectory, and Specific Ask (i.e. the health or healthcare issue patients most wanted to focus on to help achieve their most desired activity) [23]. Each session began by reviewing the template. Because there was no a priori approach for aligning individual patient’s priorities with clinical decisions, the group engaged in emergent learning, defined as learning which arises from conversations and interactions among the participants [26]. Following collaborative learning techniques, everyone was encouraged to share their experiences and suggestions concerning patient priorities-aligned decision-making for the selected patient and other similar patients [27–29]. The group discussed challenges, posed possible solutions, and worked together to determine how best to align care options with the selected patient’s specific health priorities.

Most discussions were recorded and transcribed by a professional transcription service; detailed notes were available for the remaining sessions. This study was approved by the Human Investigations Committee at Yale University.

### Analysis

Congruent with participant observation design, the members of Patient Priorities implementation team with expertise in clinician training, behavioral medicine, and care of older adults with MMCs, participated both in the discussions with clinicians and in interpreting the discussions from which the challenges and strategies were identified [25]. Following a qualitative analytical design, two members of the Patient Priorities implementation team (DS and LD) independently reviewed and interpreted the content of the transcripts and notes from the facilitated discussions to identify decisional challenges to patient priorities care alignment and strategies to potentially address these challenges. Using the constant comparative method, they continuously compared and categorized the data to identify, and progressively refine, emerging challenges until no new decisional challenges were found [30,31]. They then consolidated, refined, and agreed on an initial set of decisional challenges [30,31]. These initial challenges were reviewed and modified by all members of the Patient Priorities implementation team, described above, until there was consensus that they reflected the clinical scenarios and discussions. The same process was used to identify strategies to address these challenges and to help clinicians align decision-making with each patient’s health priorities. We followed the COREQ standards for reporting qualitative research findings [32,33].

## Results

The Patient Priorities implementation team and clinicians reviewed and discussed 35 patient scenarios over the 21 sessions. These sessions included 10 face-to-face (five with PCPs; four with cardiologists; and one with both groups) and 11 telephonic (six with PCPs, two with cardiologists; and three with PCPs and cardiologists) case-based group discussions. The patients discussed ranged from 67–98 (median, 78) years old; 75% were female; all were Caucasian. All had at least five chronic conditions. The number of active problems listed in patients’ EHR ranged from 7–66 (median, 19). Patients received from 5–16 (median, 10) prescription medications. Descriptions of the 35 patients discussed are displayed in S1 Table. Sociodemographic and function information, chronic conditions, active problems, and medications were ascertained from their EHRs. Health outcome goals and healthcare preferences were ascertained from their health priorities identification process.

Several challenges and strategies were identified from review and interpretation of the discussion sessions. Rationales were also identified for the strategies as were approaches for implementing the strategies.

### Decisional challenges (Table 1)

**Table 1:** Challenges in aligning clinical decisions with patients’ health priorities among older adults with multiple chronic conditions.

Challenges	Representative Quote
**Uncertainty, complexity, and multiplicity make decision-making difficult**	
No obvious best decision; not knowing where to start as there was so much going on	*“This is a complicated lady*, *she’s got so many comorbidities*. *There’s too many parts of this puzzle that we don’t have enough information about it to make smart recommendations*.*” (PCP about Patient # 6)* *“… His diabetes is one thing*, *but what contributions his aortic stenosis and/or medications may be having*? *He’s on a lot of high blood pressure medications…” “He’s got like 30 things wrong and he’s on a ton of medications*.*”* (Cardiologist about patient # 15 for whom lightheadedness impedes his goal to go to casino and grocery shop)
Often no single identifiable or remediable symptom	*“He has symptoms that can be related to a lot of different things…”* (PCP about patient # 28 who complains of fatigue and lightheadedness) *“…our discussion revolved around these different problems*, *which are difficult to tease out*. *How much of her fatigue and shortness of breath is [due to] obesity*, *how much is DJD*, *how much depression plays into it…*.?*”* (PCP about Patient # 34)
**Differing perspectives on what matters most**	
Patient prioritizes current discomfort or treatment burden; clinician prioritizes risk of future event	*“I think that if we can explain to patients the reason why they’re on Coumadin*. *The risk of stroke versus risk of bleeding … strokes*, *even at 92*, *are oftentimes in the setting of afib debilitating*.*”* (Cardiologist about patient # 2)
Clinicians vary in their views of the relative importance of information and which treatments are most likely to help patient	*“He wants to leave her on the Coumadin… I approached from “what matters most to her” and he quoted studies to me… we can’t get any consensus* …” (PCP about patient # 2) Cardiologist‘s perspective concerning patient # 2 is noted above.
**Difficulty switching from disease guidelines to patients’ priorities as the focus of decision-making (even knowing patients’ priorities)**	
Uncertainty whether treatment benefits reported in disease guidelines apply to this population	*“We have established a bunch of fairly specific*, *rigid guidelines for the care of patients…I don’t know if it helps*” (PCP about decision-making with patients with MCCs) *“I’m not sure how many 90-year-olds were in the studies…*. *I would venture that there probably is a little more uncertainty in a 90-year-old*…” (Discussion concerning Patient # 2)
Revert to disease guideline-based decision-making despite knowing patients’ priorities.	*“…*. *He needs all of his heart medicines*. *He has class one guideline evidence supporting his medications*.*”* (Cardiologist about patient # 8) *“I didn’t talk specifically about her goals*. *My goal is to fix her blood pressure*.*”* (PCP about patient # 143)

The three categories of challenges discussed during the sessions were the uncertainty, complexity, and multiplicity of conditions and treatments; differing perspectives on what mattered most; and the difficulty switching from disease guidelines to patients’ priorities as the focus of decision-making. Other challenges mentioned by a participant, but not discussed during the sessions, included outcome goals that were too vague or not actionable; a disconnect between the outcome goals patients desire and what they are willing to do to achieve these outcomes; and the impossibility for health priorities to fit every potential medical decision. Only the three categories of challenges that were discussed among the participants are included in Table 1.

The first category of challenges centered around the uncertainty, complexity, and multiplicity of health conditions and treatments—inherent to the presence of MCCs—that make decision-making difficult even with knowledge of individuals’ health priorities. Clinicians commented on not knowing where to start and not having an obvious best decision, *“There’s a lot of variables to account for*. *He’s got like 30 things wrong and he’s on a ton of medications*.*”* Dealing with multiple symptoms in the face of multiple conditions and treatments—what caused them, how to eliminate them, and which symptoms were most limiting—was mentioned often as a source of uncertainty and complexity.

Differences in perspectives of what mattered most between patients and clinicians or among clinicians was the second category of challenges. Clinicians occasionally expressed concern if patients considered current harms and burdens of care more important than prevention of future bad events, feeling that some patients did not appreciate the importance of these events, *“I think that if we can explain to patients the reason why they’re on Coumadin*, *and perhaps some patients like numbers*. *I don’t know*, *whatever works for that patient*…” Clinicians caring for the same patient may differ in what treatments best helped patients achieve their outcome goals as suggested by one clinician, “*I talked with* [another clinician caring for the patient] a*nd he wants to leave her on the Coumadin*. *I don’t think she is going to have any benefit*. *I approached from ‘what matters most to her’ and he quoted studies to me*.*”*

The third challenge category observed was the tendency to revert to disease-based decision-making. Clinicians recognized that the need to follow disease guidelines at times impeded making patient priorities-aligned decisions. As one PCP noted, “*We have established a bunch of fairly specific*, *rigid guidelines for the care of patients…”*

### Strategies for implementing patient priorities-aligned decision-making (Table 2)

**Table 2:** Strategies for implementing patient priorities-aligned decision-making.

Rationale for the Strategy	Tips and Scripts for Using the Strategy
**Strategy 1: Start with one actionable thing that matters most to the patient**	
In the absence of an obvious best decision and in the presence of uncertainty, where else would you start except with what matter most to the patient? Adherence is likely to improve if you begin with what matters most to the patient.	The “Specific Ask” helps start patient priorities-based communication and decision-making. *“I want less back pain and dizziness so that I can*: *keep living at home and do more with my husband around the house”* (Patient # 457). Use the response to the “Specific Ask” in decision-making.
**Strategy 2: Conduct serial trials of starting, stopping, or continuing therapies. Measure effect on patient’s health priorities**	
In the face of uncertainty, serial trials, measuring success (or failure) against attainment of health priorities, helps clinicians titrate care to maximize benefit and reduce burden.	Acknowledge that there is no single right answer. Establish timelines and use patient goals as metrics of success or failure. *“We can’t always be sure what will work best for each person*, *…We can see if it [the change] helps over the next two months*. *If not*, *we will work together to try different things*.*”*
**Strategy 3: Function over symptoms (Focus on achieving activities—health outcome goals—rather than eliminating symptoms)**	
Focusing on how symptoms are interfering with meaningful activities may be more productive than trying to eliminate symptoms. This is because, as noted in challenges, it is often uncertain what is causing the bothersome symptom. Also, it is often not possible to eliminate the symptom completely. In these situations, linking treatments to the patient’s specific goal activity can guide decision-making and be effective. Focusing on activity often paradoxically improves symptoms and is a good metric for tracking whether a treatment change is working.	Focus on achieving patient’s desired activity, *“If you were in less pain (less tired*, *SOB)*, *what would you be doing more of*?*”* Acknowledge the uncertainty and that serial trials are often needed. *“There are several possibilities… we can’t be sure what is causing (symptom)*. *A good place to start is (proposed change)*. *We’ll see if it helps you (desired activity)*.*”* *“The nice thing is you have something to guide against- is the lightheadedness sufficiently resolving to do these activities*.*”* (PCP about patient # 423)
**Strategy 4: Priorities-based communication (Use patient’s health outcome goals and healthcare preferences to discuss care).**	
Focusing communication on patients’ priorities encourages decision-making based on these priorities. Adherence is likely to improve if recommendations are tied to meaningful outcomes for patients.	Link recommendations to goals and care preferences, *“I know you don’t want procedures but the valve procedure may help the tiredness that keeps you from walking your dog and may help decrease your medications*. *You said those things are important*.*”*
**Strategy 5: Negotiate a shared decision (when there are differences in perspectives)**	
Individuals may have different perspectives and use different information to make decisions. There is no one best answer for patients with multiple conditions and variable priorities. In the face of uncertainty, patients’ priorities are the obvious unifying target of decision-making.	Agree on information that informs decision (i.e. patient’s priorities, intervention burden, all chronic conditions, life situation, health trajectory). When patient-clinician differ, present estimates of benefits and harms, expressed in the context of patient’s priorities. Be realistic about absolute benefit (often modest). Accept that older adults appropriately may value current health over future events. When clinicians differ, use collaborative negotiations, brainstorm alternatives. and agree on compromise solution, *“We could try a three-month trial off Coumadin… does she get to her dining room*? (Cardiologist and PCP about patient # 39)

The first strategy that emerged to address these challenges was to start with what was most important to each patient. Prioritizing one actionable thing that is most important to the patient helped simplify decision-making. This operationalizes patient priorities into one focused “Specific Ask (One thing): “*The one thing about my healthcare I most want to focus on is* X so that I can do (desired activity) *more often or more easily*.” The behavioral medicine expert (LD) noted that starting with patients’ priorities helps engage patients as active partners in their care while encouraging clinicians to focus on what was important to the patient. The Patient Priorities implementation team felt the Specific Ask served as a means of linking patients’ priorities with decision-making by aligning the outcome patients most desired with the health or healthcare issue they considered the key barrier to achieving the outcome.

Conducting serial trials was a second strategy identified. Participants agreed that serial trials of starting, stopping, or continuing various interventions was the most practical strategy for ongoing decision-making given the complexity and uncertainty involved. Success or failure of the trials should be defined by whether patients achieved their health outcome goals or healthcare preferences, *“There are several things that we could do…We will work together to try different things if that is ok with you*.*”*

The third strategy was focusing on achieving desired activities (patient’s health outcome goals) rather than eliminating symptoms. The uncertainty of causality and low likelihood of complete alleviation of symptoms often makes the achievement of activities a more successful clinical strategy. Increased participation in meaningful activities is also a motivator for patients and a good metric for tracking whether a treatment change is working, *“If you were in less pain (less dizzy*, *not so tired*, *weren’t so depressed)*, *what would you be doing more of*?*”*

Basing communication between patients and clinicians and among clinicians on patients’ priorities was a fourth strategy that evolved. Reminders that patient’s health priorities, rather than disease guidelines alone, should be the focus of communication helped move decision-making to patient’s priorities by considering the potential benefits, harms, or burdens of diagnostic and treatment options within the context of these priorities. “*Would you be willing to try the CPAP for a week and see if it helps with your fatigue*? *If it helps with your fatigue*, *you may be able to walk more with your wife*.*”*

The fifth strategy focused on arriving at shared decisions based on patients’ priorities when there were differences in perspectives between clinicians and patients or among clinicians. Steps that contribute to shared decisions consistent with patients’ priorities include agreeing on the information that informs the decision; realistically estimating treatment benefit; using collaborative negotiations including brainstorming alternatives; and accepting patients’ decisions if they understand the benefits and harms (Table 2).

The implementation team concurred that the identified challenges could be addressed by various combinations of the strategies. Conversely, each strategy addresses one or more challenge.

## Discussion

Through discussions among clinicians and the Patient Priorities implementation team, we identified challenges to aligning clinical decisions with patients’ health priorities as well as feasible strategies for translating patients’ health priorities into decisions and care. The challenges can be addressed by several of the strategies and, in turn, each strategy addresses one or more challenge. For example, all five strategies facilitate decision-making in the face of the uncertainty and complexity inherent in the care of older adults with multiple coexisting conditions and variable priorities. Similarly, all the strategies help get patients and their clinicians on the same page when they start with differing perspectives. Clinicians facing difficulty switching from disease guideline—to patients’ health priorities—aligned decision-making can start with one actionable thing that matters most to the patients and conduct serial trials using patient’s health outcome goals and healthcare preferences in communications with patients and other clinicians. Taken together and applied in appropriate situations, the decisional strategies can guide implementation of patient-centered care, particularly for persons with MCCs [16].

Other approaches for helping clinicians focus on achieving patients’ goals and preferences have been developed [15,17–20, 34–37]. These approaches target specific health problems or patient populations [15,17–20,34,35], or provide limited guidance in how to align decision-making with patients’ goals and preferences [36,37]. Patient Priorities Care builds on this earlier work to encompass all persons with multiple conditions and provide guidance in how to link all types of available healthcare to patients’ priorities.

A strength of the current work is its foundation in empiric observation and collaborative, emergent learning [25–29]. Problem-solving evolved from discussion of actual clinical scenarios, with their inherent complexities and nuances, ensuring our strategies and tactics were based on situations as they occur in practice. Both strategies and challenges were identified from multiple perspectives including primary care providers, specialty clinicians, and experts in clinician training and patient and clinician behavior. The strategies that emerged were those considered effective and feasible from all these perspectives. Some of the strategies recommended were based on experience from other fields. The focus on functional activities rather than solely relief of symptoms, for example, comes from the pain management field [38,39]. Negotiating a shared decision when there are differences in perspectives is a collaborative negotiating technique in the business field and is also an underpinning of collaborative and shared decision-making in healthcare [40,41]. Starting with one actionable thing that matters most and conducting serial trials were practical recommendations for addressing the uncertainty and complexity of care involving older adults with multiple conditions.

Prior to starting the pilot, the participating clinicians raised concerns about urgent decisions not covered by patients’ health priorities and about patients’ identifying unrealistic goals given their health status and trajectory. However, during the pilot, the clinicians concurred that the process the patient went through in setting health priorities helped frame big decision discussions, such as one patient with heart failure facing a decision about placement of an implantable cardioverter defibrillator. Because identifying realistic, achievable goals was part of the process, unrealistic goals were rare. One example was a 90+ year old woman with advanced heart failure who wanted to play nine holes of golf, walking the course. Using collaborative negotiations and priorities-based communication, the patient agreed to previously declined testing to better identify potential treatments that might help her achieve her priorities. She also agreed to a more realistic goal of using a golf cart and playing fewer holes.

Of necessity, the strategies are general and must be adapted for each clinical situation. As experience with this approach to decision-making increases, additional challenges and strategies may emerge for aligning healthcare with patients’ health priorities. Nevertheless, it was reassuring to observe that a few strategies could be used to address wide-ranging issues faced by patients with multiple conditions and by the clinicians caring for them.

There are additional limitations to this study. The challenges and strategies reflect observation of a single group of clinicians in a single setting involving a discrete number of patients. While valid within the context of the population and setting of the current study, our findings require replication in additional settings with diverse groups of patients. The inclusion of other specialties who care for older adults may reveal additional challenges and strategies. We have not yet pursued widespread uptake of these strategies nor have we determined the effect such strategies and tactics would have on patient outcomes. Evidence supporting the benefits of current diagnostic, preventive, therapeutic and other interventions for individual health priorities remains lacking. As research focuses directly on the outcomes that matter to persons with multiple conditions, this evidence will increasingly emerge.

Identifying feasible strategies suggest that patient priorities-aligned decision-making may be possible in clinical practice. Indeed, clinicians noted using some of these strategies before participating in this pilot. The current work suggests that implementing these strategies systematically and explicitly may offer an approach to decision-making in the face of complexity and uncertainty for persons with multiple conditions, perhaps helping to reduce intervention burden and increase likelihood of achieving patients’ most desired outcome goals.

The Patient Priorities Care initiative is continuing. In parallel to our work with clinicians, we are guiding patients in how to identify and communicate their health priorities [23]. Ongoing work also includes evaluating the effects of identifying patients’ priorities and using them in decision-making on patient, clinician, and health system outcomes. Once we have further developed and tested the Patient Priorities Care approach, including the strategies identified in this study, we will engage additional clinical practices and sites to assess acceptance and uptake. The eventual goal is the development and dissemination of an approach to decision-making and care that maximizes benefits that matter to individuals with multiple chronic conditions while minimizing harm and intervention burden.

**IRB APPROVAL**: Yale Institutional Review Board (IRB) approved this study prior to data collection. The IRB approved verbal consent to participate in this study. All patients provided verbal consent to participate, share the data collected in the study with the investigators and publish without identifying information. Several of the authors, the Yale Chief HIPAA officer, and the Executive Director at the participating primary care practice reviewed the information included in the Supporting Information to ensure individuals could not be identified based on personalized details.

## Supporting information

S1 File Example of completed health priorities template. (DOCX)

S1 Table Characteristics, health priorities (health outcome goals and healthcare preferences) of patients selected for the facilitated discussion sessions*. (DOCX)
