# Supplementary material for: Challenges and strategies in patients’ health priorities-aligned decision-making for older adults with multiple chronic conditions
Source: PLoS One. 2019 Jun 10;14(6):e0218249. doi: 10.1371/journal.pone.0218249 (PMC6557523; doi:10.1371/journal.pone.0218249)
Supplement: S1 File — (DOCX) [file pone.0218249.s001.docx]

**S1.** **Example of completed Health Priorities Template**:

**Patient Priorities Care: Health Priorities Template**

**Current Function and Support**:

80+ yrs, Resides in own home with spouse, independent with ADLs

**Health trajectory** (Current understanding of how health will likely change over the next few years):

“I want to just get up in the morning”

**Matters most (Values)**:

Independence and Mobility—live in own home, be able to get up and get out

Connection with others—children, grandchildren, husband

**SMART Health Outcome Goals**

1. I want to keep living in my own home, be able to get up and get dressed—back pain and dizziness makes this difficult

2. I want to be able to maintain my own home—back pain and dizziness are a current barrier to this

3. I want to continue to keep planning and cooking meals—back pain makes this difficult

**Helpful care:** The medications, self-management tasks, clinical visits, tests, or procedures, that I think are helping me most with my health goals and I can do them without too much difficulty

1. I do bed exercises to help my back
2. I check my blood sugar once a month maybe, only once in a while
3. My eye drops are helping my Glaucoma, I want to keep seeing

**Difficult or bothersome care**: The medications, self-management tasks, clinical visits, tests, or procedures that don’t think are helping my goals and are bothersome or too difficult for me. I would like to talk with my doctor about whether these are helping my goals. If not, can I stop them or cut back? If they are helping, is there a way to make them less bothersome or less difficult?

1. I’ve had too many surgeries, I have several screws in my back
2. I am getting lightheaded and I don’t know if it is because of some of my medications or my diabetes

| **Specific ask (One Thing):** The one thing about my healthcare I most want to focus on is (*fill in a health problem that you think is keeping you from achieving your health outcome goal OR the healthcare task that is most bothersome or difficult*) so that I can do (desired activity) more often or more easily.  I want less back pain and dizziness **so that I can** keep living at home and do more with my husband around the house |
| --- |
