# Supplementary material for: Challenges and strategies in patients’ health priorities-aligned decision-making for older adults with multiple chronic conditions
Source: PLoS One. 2019 Jun 10;14(6):e0218249. doi: 10.1371/journal.pone.0218249 (PMC6557523; doi:10.1371/journal.pone.0218249)
Supplement: S1 Table — (DOCX) [file pone.0218249.s002.docx]

| **S1 Table. Characteristics, health priorities (health outcome goals and healthcare preferences) of patients selected for the facilitated discussion sessions*** | | | | | |
| --- | --- | --- | --- | --- | --- |
| **No.** | **Characteristics, Conditions; Active problems; Medications^a^** | **Health Outcome Goals^b^** | **Healthcare Preferences^b^** | | **Specific Ask^c^** |
|  |  |  | **Helpful and doable care** | **Bothersome, unhelpful, or unwanted care** |  |
| 1 | 70+ y.o. F; independent with ADL  Chronic conditions: asthma; sleep apnea on CPAP; arthritis (knees, hands); carpal tunnel syndrome, asthma, hypertension, hyperlipidemia,  depression, hypothyroidism,  obesity, CVA,  Active problem list: 35 problems  13 prescription medications: Albuterol; aspirin; clopidogrel; Zyrtec, Losartan, Lovastatin; Metoprolol, Levothyroxine, Voltaren gel, Ventolin, Meclizine  Niacin; ProAir; Vitamin D | Continue to:  - do yoga at the senior center each week  Be able to:  walk around park each week  Arthritis pain is barrier | The hand dip  I use the tension ball each day  I am taking three Tylenol Arthritis each night | It bothers me that I can’t get what I want [NSAID} to help the pain in my hands  I don’t know if I want a knee replacement  I am not going to wear compression stockings | The arthritis pain in my hands**, so that I can** keep going to the senior center and participating in classes. |
| 2 | 90+ yo F; gets help with ADLs  Chronic conditions: Atrial fibrillation, arthritis, hypertension, depression, hypothyroidism,  Active problem list: 7 problems  7 prescription medications: digitalis, furosemide, hydrochlorthiazide,  Escitalopram  Vitamins D  levothyroxine,  warfarin | Be able to:  -attend meals in the dining room at her building daily  - weekly trips to do her shopping with her aide  Balance; back and knee pain; fear of falling and bleeding and bruising are barriers | Tylenol helps with arthritis but “I’m not sure how much I can take.”  Hearing aids and scooter are helpful in getting outside apartment  Willing to have INR draws (at retirement community) | Fearful of trying new medications for arthritis pain “my oxycodone and Celebrex made me dizzy  Not able to do seated exercise classes because of knee pain  Bleeding and bruising with warfarin is making her fearful of physical activity | I need help with my balance and the pain in my back and legs **so that I can** walk to the dining room and visit my friends |
| 3 | 70+ y.o. F; walks with cane  Chronic conditions: Anxiety; CAD; depression; GERD; hyperglycemia; spinal stenosis; supraventricular tachycardia  Active problem list: 23  6 prescription medications: Aspirin; atenolol; Atorvastatin; Gabapentin; Nitrostat; Sertraline | Be able to:  -go to grocery store weekly.  Leg pain a barrier | Trying gabapentin for pain  Willing to use cane  Physical therapy  Wants to go back on statin | Lipitor may be causing pain  Fearful of new medications-experiences many adverse effects  Does not want surgery  Epidural and sacroiliac injections did not help | NA |
| 4 | 80+ y.o. F; independent with ADLs  Chronic conditions: Anxiety; arthritis; DVT; GERD; hypertension; hip fracture; irritable bowel syndrome.  Active problem list: 60 problems  3 prescription medications: Amlodipine; Vitamin B12; Vitamin D | Continue to:  - go to restaurant daily to socialize and have lunch  Be able to:  - be a greeter at her club  IBS and PT are barriers | Willing to try restaurants with healthier food choices  Pleased with physical therapy, PCP and GI clinicians | Experiencing dizziness, uncertain if due to medications  Not ready for assisted living | NA |
| 5 | 70+ y.o. M; uses cane occasionally  Chronic conditions: Atrial fibrillation; BPH; CAD; CKD Stage 3; congestive heart failure; depression; DM Type 2; diabetic neuropathy; hypertension; hyperlipidemia; insomnia; obesity; hyperparathyroidism; Vitamin D deficiency  Active problem list: 39  12 prescription medications: Allopurinol; aspirin; buspirone; calcitriol; carvedilol; colchicine; coumadin; ferrous sulfate; furosemide; mirtazapine; nateglinide; pravachol | Continue to:  - go to flea markets on weekends.    -do service projects in the community  dyspnea and fatigue are barriers | Gout medications shorten duration of attacks  Exercises given by chiropractor for back pain  INR draws every 3-4 weeks  Cardiologist and frequent visits with PCP | Does not wish to make other dietary changes  Not willing to check blood sugar more often, only does it “when my wife makes me” | NA |
| 6 | 80+ y.o. F; lives alone; uses cane or walker  Chronic conditions: Arthritis; CVA; CKD stage 3; depression; DM Type 2; gout; hypertension; hyperlipidemia; hypothyroidism; peripheral neuropathy; urinary incontinence  Active problem list: 34 problems  10 prescription medications: Amlodipine; clopidogrel; levothyroxine; Linzess; lisinopril; Lyrica; Morphine ER; omeprazole; Pristiq; Vitamin D | Be able to:  -walk her dog around the property twice daily  -have dinner with her daughter weekly | Ask her son install hand rail  Neck exercises daily  Prepour medications | Would like to decrease blood pressure medications if possible  Constipation a problem | I want to be less dizzy and unsteady in the morning **so that I can** keep going for walks to see my neighbors and take care of my dog |
| 7 | 80+ y.o. M; ambulates with rolling walker  Chronic conditions: Atrial fibrillation; BPH; DM Type 2; chronic diarrhea; GERD; hyperlipidemia; hypothyroidism; hypotension; PVD; Vitamin B12 deficiency; Vitamin D deficiency  Active problem list: 17 problems  16 prescription medications: Amiodarone; Atorvastatin; digitalis; fludrocortisone; gemfibrozil; hydrocodone; ferrous sulfate; levothyroxine; magnesium; midodrine; Omeprazole; sitagliptin; Pradaxa; Tamsulosin; Vitamin B12; Vitamin D | Be able to:  -Go to lunch on with wife  - visit with grandchildren once per month  - do cooking projects with wife weekly  Back pain and dizziness are barriers | Referral to pain clinic  Have wife manage medications;  written log with check boxes for medications  Use electric lift chair, rolling walker  PCP and cardiology | Hydrocodone-acetaminophen not helping pain  Uncertain if medication is contributing to joint pain  Declines doing blood sugar checks | NA |
| 8 | 70+ y.o. M; independent with ADLs  Chronic conditions: Atrial fibrillation; congestive heart failure; depression; hyperlipidemia; hypertension; morbid obesity; nephrolithiasis; sleep apnea on CPAP  Active problem list: 16 problems  7 prescription medications: Atorvastatin; Citalopram; Jantoven; Lisinopril; diltiazem XL; trazodone; warfarin | Continue to:  Be able to:  - walk and climb stairs more easily  - be more active with wife.  Fatigue, back pain, pannus are barriers | Blood pressure medications and vitamins are helpful and “they are not that bad.”  Willing to stay on warfarin and have INR monitored  “Heart operation” helped with fatigue and SOB  Cataract surgeries were helpful.  PCP and cardiologist | Not willing to take medications for back pain (including Tylenol), afraid of addiction and “losing my marbles.” | NA |
| 9 | 70+ y.o F; independent in ADLs  Chronic conditions: atrial fibrillation; anxiety; hyperlipidemia; hypertension; hyperglycemia; overweight  Active problem list: 13 problems  10 prescription medications: Aspirin; calcium; glucosamine; lorazepam; lovastatin; metoprolol; rosuvastatin; verapamil; Vitamin B 12; Vitamin D | Be able to:  -go out to dinner with her husband twice per week  back and right knee pain are barriers | Will consider knee surgery if recommended  Manages own medications  Exercises art gym  PCP and cardiologist | Intermittent dizziness- uncertain if medications are contributing  Increased muscle pain uncertain if medications are contributing | NA |
| 10 | 70+ y.o. F; assists husband with ADLs  Chronic conditions: Arthritis; hypertension; cataract; depression; GERD; obesity; restless leg syndrome; sleep apnea on CPAP; urge incontinence  Active problem list: 34 problems  10 prescription medications: Advair; diltiazem;  escitalopram; ferrous sulfate; hydrochlorothizide; omeprazole; pramipexole; valsartan; vitamin B12; vitamin D | Be able to:  - walk around the neighborhood each week  -work in her yard each week  Lack of motivation and knee pain are barriers | Escitalopram “feels it’s working”  Elevates legs  Visits to PCP; Cardiologist; Orthopedist | Afraid to have Arthroscopy of L knee  Dieting to lose weight too hard | NA |
| 11 | 90+ y.o F; partially dependent with all ADLs  Chronic conditions: GERD, CKD Stage 3, CAD, depression, arthritis, hypertension, hyperlipidemia, macular degeneration, urinary incontinence  20 problems in active problem list.  10 prescription medications: Ranitidine, aspirin, Cartia, Gabapentin, Lorazepam, Lumigan eye drops, Lutein, Magnesium, Calcium &Vitamin D, Pantoprazole, Tylenol | Continue to:  - go out weekly to get my hair done  -go to church each week  Be able to:  - help my daughter with housework and putting dishes away  Difficulty walking and imbalance are barriers | Injection in my eye for the macular degeneration  Pedialyte to help with my electrolyte imbalance  Gabapentin helps so much, my leg used to jump and I would get an electric shock pain | Tramadol knocks me out  Lorazepam is not helpful,  Not have to depend on depends | I want to walk better and have better balance **so that I can** help my daughter more with housework |
| 12 | 80+ y.o. F; lives in own home  Chronic conditions: Arthritis; CKD stage 4; DM; history of malignant melanoma; gout; hypertension; obesity  Active problem list: 10 problems  7 prescription medications: Allopurinol; calcitriol; Claritin; hydrochlorothiazide; lisinopril; metoprolol; vitamin D | Continue to:  -work in her garden each day  - drive to babysit grandchildren each week | Renal diet  Increase in blood pressure medications  Limit use of NSAIDs | Interferon caused depression “I refused to go back on it, I don’t want to live that way  Recommended foot surgery; concerned about decreased postoperative mobility | NA |
| 13 | 80 y.o. M; independent in ADLs  Chronic conditions: Arthritis; CKD stage 3; hypertension; bladder cancer; colon cancer; hyperlipidemia; obesity; legally blind; Vitamin D deficiency  Active problem list: 16 problems  11 prescription medications: amlodipine; aspirin; atorvastatin; lisinopril; doxazosin; vitamin D; ICaps AREDS; latanoprost; hydrochlorothiazide; metoprolol; ProAir inhalant | Continue to:  - go out to breakfast each day  No barriers  Be able to:  - fish each week  Lymphedema care a barrier | Cane has stopped me from some falls  Denies dizziness or GI problems from 3 blood pressure medications | Declined cataract removal | NA |
| 14 | 70+ y.o. M; partially dependent in ADLs  Chronic conditions: Arthritis; adhesive capsulitis shoulder; aortic stenosis; CKD stage 2; constipation; DM type 2; anxiety; hypertension; hyperlipidemia; inguinal hernia; sleep apnea; nephrolithiasis; peroneal muscle atrophy; GERD; urinary retention  Active problem list: 24 problems  8 prescription medications: Aspirin; CoQ10; famotidine; metformin; metoprolol; Miralax; simvastatin; trazadone | Continue to:  -get out each day with his friends to have coffee and to play cards  -go to the gym each day  -volunteer at the hospital once per week | Reduction in simvastatin to 3 times per week is helping, decreased muscle pain  Trazodone is helping with sleep  CPAP is “a pain” but it helps | Tamsulosin did not help | NA |
| 15 | 80+ y.o M; independent in ADLs  Chronic conditions: Aortic stenosis; abdominal aortic aneurysm; DM  34 active problems listed in EHR  7 prescription medications: Amlodipine; aspirin; Atorvastatin; Flomax; Isosorbide; Ranolazine; Insulin | Be able to:  - go to the casino each week  - take the bus to visit family  - drive to the grocery store each week  Lightheadedness is barrier | Personal emergency response system  Self-administer insulin | Blood sugar testing 6-7 times per day due fear of passing out  Insulin administration four times per day  Aortic valve replacement, “I don’t know if this will be helpful or not” | Be less lightheaded and short of breath **so that I can** go to the casino and grocery shop |
| 16 | 70+ y.o. F; independent with ADLs  Chronic conditions: Anemia; anxiety; atrial fibrillation; GERD; hypertension; multiple myeloma; overweight  Active problem list: 14 problems  8 prescription medications: amiodarone; calcitriol; doxazosin; losartan; metoprolol; revlimid; warfarin | Continue to do:    -Yoga 3-5 times per week  - outdoor activities  Bone pain a barrier | Immunomodulator for cancer treatment  Blood work monthly  Occasional opioid helps pain | Exercises that bother her joints | NA |
| 17 | 80+ y.o. F; independent with ADLs  Chronic conditions: Atrial fibrillation; arthritis; constipation; congestive heart failure; diverticulitis; hyperlipidemia; hypertension; degenerative disc disease; pulmonary hypertension; urinary incontinence; Vitamin D deficiency  Active problem list: 18 problems  7 prescription medications: Aspirin; folic acid; hydrochlorothiazide; metoprolol; olmesartan medoxomil; pravastatin; Vitamin D | Continue to:  - volunteer three times per week at senior center  Be able to:  -garden once per week  Pain in hands is barrier | Copper Arthritis Gloves  Elevates feet  Takes medications at night | Even if surgery could help my foot, I don’t want to go through it | Less muscle pain in my hands **so that I can** garden once per week |
| 18 | 80+ y.o. F; independent in ADLs  Chronic conditions: atrial fibrillation; alcohol abuse; arthritis; past breast cancer; GERD; hypertension; hypothyroidism; low back pain; macular degeneration; gait disturbance  Active problem list:18 problems  9 prescription medications: amiodarone; amlodipine; atenolol; furosemide; levothyroxine; lisinopril; potassium; tramadol; xarelto | Continue to:  - go to Florida for the winter months  Be able to:  - go to the casino 2-3 times per year  - do more of my own housework and even some gardening  Back pain is barrier | Tramadol  Use cane  No dizziness from medications | Pain center treatments  Over the counter pain remedies | NA |
| 19 | 90+ y.o. F, has help with ADLs;  Chronic conditions: DM type 2, Stage 4 CKD; atrial fibrillation; congestive heart failure; hypertension; hyperlipidemia; hypothyroidism  16 problems listed in active problem list  12 prescription medications: Diltiazem; furosemide; insulin; lisinopril; metoprolol; pravastatin; Preservision AREDS, Vitamin D; warfarin | Continue to:  -participate in dinners and card games  -go out shopping with my companion each week  Be able to:  - do more of my own housework  Weakness, pain, and diarrhea are barriers | INR blood work once per month; recently increased to weekly  Rollator walker | Doesn’t wish to see more specialists  Wants “no surgery of any kind” | I can’t go where I want to as much because of this diarrhea |
| 20 | 80+ y.o. F; partially dependent in ADLs  Chronic conditions: arthritis; amaurosis fugax; anemia; atrial fibrillation; gait disturbance; low back pain; CAD; depression; GERD; hyperlipidemia; hypertension; sacroiliitis  Active problem list: 29 problems  10 prescription medication: amlodipine; atenolol; atorvastatin; Vitamin B12; clopidogrel; Eliquis; glucosamine; isosorbide; lisinopril; pantoprazole | Be able to:  -go for a walk to the end of her street  - get in and out of a car to go out to lunch with friends  back pain is barrier | Would like to try a back brace  Blood draws for INR | Would like to take less medications if possible  Not using durable medical equipment for walking due to cost  Tylenol Arthritis does not help with back pain | I would like to have some relief of lower back pain that shoots down my legs **so that I can**  walk to the end of my street and get out of the car by myself |
| 21 | 80+ y.o. M; gait is unsteady but no falls.  Chronic conditions: Aortic stenosis; constipation; DM Type 2; gait disturbance; hypertension; Parkinson’s disease; seizure disorder; sick sinus syndrome; urinary incontinence  Active problem list:16 problems  7 prescription medications: amlodipine; aspirin; carbidopa-levodopa; levetiracetam; metformin; miralax; valsartan | Be able to:    -walk up the stairs  - go to church  Unsteady gait and lack of energy are barriers | Constipation is better, Miramax works. | I want to know if the medications are making my vision worse. | I want to have less incontinence at **night so that I can** have more energy to go out and go to church and be awake to read |
| 22 | 70+ y.o. F; independent ADLs  Chronic conditions: Anxiety; arthritis; Crohns disease; GERD; glaucoma; hyperlipidemia; hypertension; Vitamin B12 deficiency  Active problem list: 12 problems  15 prescription medications: aspirin; buspirone; calcium; clonazepam; diltiazem; dorzolamide-timolol; gabapentin; omeprazole; pravastatin; quetiapine; restasis; valsartan; venlafaxine; Vitamin D3 | Continue to:  - go the senior center 3-4 times per week  -go out to lunch with friends once per week  No barriers  Be able to:  - walk within her neighborhood once per week  -participate in a yoga class at the senior center  Lower back pain is barrier. | Physical therapy has helped the pain on my left side,  Want to discuss options to manage symptoms of costochondritis | Would like to be taking less medications, “this pile of meds is not an appealing breakfast”  I don’t know if my Pravastatin is causing some of my muscle pain  “I don’t know why I am taking two blood pressure medications, I want to know if I really need to” | NA |
| 23 | 70+ y.o F; lives alone, independent with ADLs  Chronic conditions: Arthritis; Alzheimer’s dementia; gait instability; hearing loss; arthritis; hypertension; obesity; hyperlipidemia diplopia; GERD; spinal stenosis  46 problems listed in active problem list  12 prescription medications, Atenolol, Atorvaststin, calcium; Flecainide, Flovent; Lansoprazole, Namenda, Pantoprazole, Flovent inhaler, ProAir inhaler, Voltaren gel, Zyrtec | Continue to:  - live alone  - work around the house”  Balance, dizziness, and memory loss are barriers | Physical therapy for dizziness and balance  Taking my blood pressure medications with food | I don’t know if I want knee surgery  I hate medications, I was told by the pharmacist that the yellow dye in the capsule may be causing dizziness | I want to check if I have any medications that are causing side effects **so that I can** continue living a normal life on my own as long as possible |
| 24 | 65+ y.o F; lives alone; independent with ADLs  Chronic conditions: hypertension; low back pain; sleep disorder; hypothyroidism    Active problem list: 3 problems listed  5 prescription medications: Lisinopril; levothyroxine; hydrochlorothiazide; potassium; trazadone | Continue to:  - travel to Florida to stay with my daughter for the winter  Be able to:  -walk with friends  Pain is a barrier | Trazadone helps me sleep  Watch what I eat and diet | “I get this electric shock pain in my hands, I don’t know if my meds are causing this”  “I get hypoglycemic, I get weak and shaky I don’t know if I’m taking too much of something”  “I don’t want to take a lot of medications” | I want the pain in my hands to get better **so that I can** make crafts and sell them to have spending money each month |
| 25 | 70+ y.o. F; lives with husband; partially dependent with ADLs  Chronic conditions: Aortic stenosis; COPD; CAD; gait disturbance; GERD; hearing loss; obesity; hypertension; hypothyroidism; hyperlipidemia; sleep apnea; restless leg syndrome; rheumatoid arthritis; urinary incontinence  Active problem list: 33 problems  14 prescription medications: Aggrenox; aspirin; Breo Ellipta; folic acid; potassium; levothyroxine; methotrexate; metoprolol; oxycodone; stool softener; triamterene; hydrochlorothiazide; Vitamin D; Zetia | Continue to:  - visit daughter at her home  Be able to:  - go out to dinner with my husband  Frequent urination is a barrier | Nebulizer when I need it  Physical therapy  Aspirin cream for my hand | “I don’t know if Zetia is causing this muscle pain”  “The water pill is making me pee a lot”  Medications are causing dry mouth | I would like to take less medications **so that I can** have less side effects like dry mouth and muscle pain |
| 26 | 80+ y.o. F; lives alone; independent with ADLs  Chronic conditions: Arthritis, atrial fibrillation, dysphagia, hypertension, hypothyroidism, hyperlipidemia, hearing loss, mitral and aortic valve disease, osteoporosis  20 problems on active problem list  11 prescription medications: Calcium, Claritin, Glucosamine, levothyroxine, Metoprolol, Niacin, Pantoprazole, Probiotic, Verapamil, Vitamin D, Warfarin | Continue to:  - help my sister  - maintain my own house, I have a hard time picking something up off the floor  -have dinner with my children and grandchildren each week  Knee pain is a barrier | “Cutting back on the Metoprolol has helped me feel less tired”  “I have atrial fibrillation and an enlarged heart so I know why I have to take the heart medications”  “How much Glucosamine can I take; I take 2 tabs once a day” | “I want to eat greens but they don’t want me to”  “I get headaches from statins so I won’t take them”  “Tylenol PM didn’t work so why take it?”  “My pain is worse since the steroid injections” | I would like less knee pain **so that I can** get around better and fall less |
| 27 | 70+ y.o. M; independent with all ADLs  Chronic conditions: Atrial fibrillation, arthritis, hypertension, hyperlipidemia, BPH, GERD, back pain, migraine headaches  Active problem list:  11 prescription medications: butalbital, doxazosin, Flecainide, Irbesartan, Meloxicam, Methocarbamol, Omeprazole, Simvastatin, Tramadol, Verapamil, Xarelto | Continue to:  -volunteer each week  -go to the senior center for exercise  Dizziness is a barrier | Taking blood thinner because they found 3 blockages in my lungs  I walk a lot and watch what I eat | “I did cut back on the specialists that I see”  “I would like to cut back on the Flecainide,  The Flecainide makes me dizzy but I am willing to have dizziness to prevent a stroke”  “If my cholesterol numbers are good why can’t I cut back on the statin?”  “I am taking less Meloxicam and want to get off NSAIDs all together, I don’t want the side effects on my heart” | I want to reduce my medications **so that I can**: alleviate worry about the side effects I’m having |
| 28 | 80+ y.o. F; lives with spouse; independent with ADLs  Chronic conditions: atrial fibrillation; glaucoma; mild cognitive impairment; obesity; essential tremor; anxiety disorder; hypertension; hyperlipidemia; hyperthyroidism;  Active problem list: 10 problems  13 prescription medications: alprazolam; aspirin; atorvastatin; Benicar; CoQ10; Vitamin D; Dorzolamide; flecainide; latanoprost; memantine; metoprolol; prevident; levothyroxine | Continue to:  - go to church each week  -cook and take care of my house  Be able to:  -do drawing and ceramics again”  Arthritis pain is a barrier | “Alprazolam is helpful, I only take it when I need it”  “My husband puts my eye drops in”  “I try to do the exercises that PT taught me every day” | “Because of the arthritis, I don’t think I can tell if I’m having more muscle pain”  “Could any of my medications be making my IBS worse? IBS holds me back from going places because I never know when I will have to go.”  “Could my medications be causing the bumps that I get on my skin, I can move them around?” | My arthritis pain in my hands **so that I can**: cook and cut up things easier, it is hard |
| 29 | 90+ y.o. M; walks with cane or walker  Chronic conditions: DM Type 2, hypertension, hyperlipidemia, depression, arthritis hypothyroidism  14 problems in active problem list  8 prescription medications: Aspirin, Atenolol, Atorvastatin, Insulin, Glipizide, Synthroid, Metformin, Venlafaxine | Continue to:  -go out to get haircut weekly  Be able to:  - go to restaurant for breakfast to see my friends  - go out to a store  Dizziness and hip pain are barriers | Check blood sugars daily  Homecare nurse visit monthly  Use cane, “I need it for hip pain” | Shaky and dizzy in the morning, “ I don’t know if my diabetes medications are too much. We already stopped the Atenolol and that helped a little.”  Don’t think I am strong enough for hip surgery  Tylenol and Cortisone injections were not helpful with hip pain | Be less dizzy and have less hip pain in the morning **so that I can** go out to breakfast and see my friends. |
| 30 | 80+ y.o. F; lives with husband, independent with ADLs  Chronic conditions: Back pain; arthritis, hypertension, hyperlipidemia, obesity, osteoporosis, syncopal episodes, DM Type 2, B12 deficiency  Active problem list: 38 problems  16 prescription medications: Acetaminophen, Amlodipine, aspirin, atorvastatin, B12, Vitamin D, Dorzolamide eye drops, Hydrocodone, insulin, Latanoprost eye drops, Lamisil cream, Methocarbamol, Metoprolol, Pantoprazole, PreserrVision, Temazepam | Continue to:  -cook meals  Be able to:  - get up and get dressed  - maintain my own home  Back pain and dizziness are barriers | Exercises to help my back  Check my blood sugar once a month maybe, only once in a while  My eye drops are helping my Glaucoma, I want to keep seeing | I’ve had too many surgeries; I don’t want another back surgery  I get light headed and I don’t know if it is because of some of my medications or my diabetes | I want less back pain and dizziness **so that I can**: keep living at home and do more with my husband around the house |
| 31 | 70+ y.o. F; independent with ADLs  Chronic conditions: DM Type 2, obesity, arthritis, hypertension, hyperlipidemia, urinary incontinence  Active problem list: 8 problems  10 prescription medications: acetaminophen, aspirin, Bisoprolol, CoQ 10, Folic acid, Insulin, Pravastatin, Valsartan, HCTZ, Vitamin D | Continue to:  - be a caregiver  -drive to church each week | I check my blood sugar every morning  The Meloxicam is helping my shoulder pain | “I do get dizzy sometimes, I don’t know if it is the diabetes medications”  “They lowered my diuretic recently and my ankles have started to swell, I don’t like that, the swelling can lead to other problems” | I would like to lower my diabetes medications **so that I can:** avoid the problems that diabetes does to you, like dizziness, vision loss and problems with feet and walking |
| 32 | 70+ y.o. F, independent with ADLs  Chronic conditions: arthritis; depression; hypertension; hyperlipidemia; CAD; GERD  Active problem list: 13  9 prescription medications: bisoprolol; CoQ10; desoximetasone; diclofenac; Nitrostat; Norvasc; omeprazole; rosuvastatin; tramadol | Continue to:  - pick my granddaughter up from the bus  - go out to dinner with friends once per month  -take my dog out each day  muscle pain in my legs is a barrier | Tramadol helps the arthritis pain  The gastric reflux is better with the omeprazole  My blood pressure medications help; last time my blood pressure was off I had three stents put in | I’m having soreness in my legs since I had an increase in the statin | I want to have more energy in the morning **so that I can** continue to walk my dog, work, and get my granddaughter off the bus |
| 33 | 70+ y.o. F; liveswith husband, independent with ADLs  Chronic conditions: atrial fibrillation, hypothyroidism, osteoarthritis, obesity, hyperlipidemia, hypertension, sleep apnea, restless leg syndrome, GERD, recent UGI bleed  Active problem list; 11 problems  7 prescription medications: Atenolol, Atorvastatin, Levothyroxine, Lisinopril, Ranitidine, Ropinirole, warfarin | Be able to:  - walk more with husband  - do ceramics again  Tiredness is a barrier | BiPaP is helpful  Blood work every month | Ropinirole causes hallucinations and nightmares | I want to be less tired **so that I can:** walk more with my husband and have more energy in the mornings |
| 34 | 70 y.o. F; lives with husband  Chronic conditions: Hypertension, depression, hyperlipidemia, insomnia, obesity, carotid artery stenosis, CKD stage 3, osteoporosis, hyperparathyroidism  Active problem list:66 problems  10 prescription medications: Allopurinol, alprazolam, aspirin, atenolol, calcium-Vitamin D, duloxetine, lisinopril, potassium, ropinirole, simvastatin | Continue to:  - go out to eat each month  - have sleep overs with my grandchildren  - bake for my grandchildren  Muscle spasms, lower back pain, constipation, and taking medications  are barriers | Check my blood sugars even though I am not a diabetic anymore  Pain management clinic  I guess the Cymbalta is helping but I would want to try going without it  Trying to lose weight to help with back pain | Stool softener caused too much cramping  Fosomax caused hair loss and muscle spasms  I want to try to get off some of the medications | I would like to try and take less medications **so that I can**: have less side effects, less to manage, maybe less constipation and more time to go for a walk |
| 35 | 80+ y.o. F;, independent with ADLs  Chronic conditions: depression, insomnia; gait disorder; hyperlipidemia; hypertension; hypothyroidism; osteoarthritis; peripheral neuropathy; restless legs syndrome; Vitamin D deficiency  Active problem list: 31 problems  14 prescription medications: amlodipine; aspirin; effexor; levothyroxine; mupirocin; mybetriq; percogesic; potassium; pravastatin; soolantra; stool softener; Vitamin D; Voltaren; zolpidem | Continue to:  -play with my granddaughter  Be able to:  - visit and go shopping with my family  Fatigue and weakness are barriers | Zolpidem is very helpful at night  Voltaren Gel is helpful with my knee pain  I start physical therapy for gait and balance next week | “I’m waking up with a hot flash, I don’t know if it is medications”  “I don’t want a knee replacement even though it is terrible going up and down the stairs, I am too worried about recovery”  “I want to know if my medications are making me feel so tired and weak | I want to get off some of these medications that are making me feel **tired so that I can** go out shopping |
| Abbreviations: ADLs, activities of daily living; BPH, benign prostatic hypertrophy; CAD, coronary artery disease; CKD, chronic kidney disease; COPD, chronic obstructive pulmonary disease; CPAP, continuous positive airway pressure; CVA; cerebrovascular accident; DM, diabetes mellitus; DVT; deep venous thrombosis; EHR, electronic health record; GERD; gastroesophageal reflux disease; GI, gastrointestinal; IBS, irritable bowel syndrome; PCP, primary care provider; PT, physical therapy; PVD, peripheral vascular disease; SOB. Shortness of breath; UGI, upper gastrointestinal  * Inclusion criteria for patients included presence of > 3 chronic conditions plus ≥ 10 medications; or saw > 2 specialists in the past year. Exclusion criteria included hospice eligibility, receiving hemodialysis, .advanced dementia, or nursing home residence).  ^a^ Age is given in decade to avoid deductive disclosure. The chronic conditions are those listed in the EHR. Active problems based on Active Problem list in the EHR.  ^b^ Health outcome goals are the health and life outcomes that patients’ desire from their healthcare. Healthcare preferences refer to the healthcare activities (e.g. medications, self-management tasks, healthcare visits, testing, and procedures) that patients find helpful and doable OR bothersome, unhelpful, or unwanted. These outcome goals and healthcare preferences were ascertained by a member of the healthcare team as part of the health priorities identification process.  ^c^ Specific Ask is the last item on the Health Priorities template. Patients are asked to identify the health or healthcare issue they most want to focus on and what desired activity improving the health or healthcare issue will help them do more often or easier. It was added during the Patient Priorities pilot Plan-Do-Study-Act cycles so several patients did not have this item on their template. | | | | | |
